# Island selection on mammalian life-histories: genetic differentiation in offspring size

**DOI:** 10.1186/1471-2148-8-296

**Published:** 2008-10-27

**Authors:** Tapio Mappes, Alessandro Grapputo, Harri Hakkarainen, Esa Huhta, Esa Koskela, Raimo Saunanen, Petri Suorsa

**Affiliations:** 1 Centre of Excellence in Evolutionary Research, University of Jyväskylä, P.O. Box 35, FIN-40014 Jyväskylä, Finland; 2 Department of Biology, University of Padova, 58/B 35121 Padova, Italy; 3 Section of Ecology, Department of Biology, University of Turku, FIN-20014 Turku, Finland; 4 Finnish Forest Research Institute, Kolari Research Station, FIN-95900 Kolari, Finland; 5 Department of Biological and Environmental Science, P.O. Box 35, FIN-40014 Jyväskylä, Finland

## Abstract

**Background:**

Since Darwin's pioneering work, evolutionary changes in isolated island populations of vertebrates have continued to provide the strongest evidence for the theory of natural selection. Besides macro-evolutionary changes, micro-evolutionary changes and the relative importance of natural selection vs. genetic drift are under intense investigation. Our study focuses on the genetic differentiation in morphological and life-history traits in insular populations of a small mammal the bank vole *Myodes glareolus*.

**Results:**

Our results do not support the earlier findings for larger adult size or lower reproductive effort in insular populations of small mammals. However, the individuals living on islands produced larger offspring than individuals living on the mainland. Genetic differentiation in offspring size was further confirmed by the analyses of quantitative genetics in lab. In insular populations, genetic differentiation in offspring size simultaneously decreases the additive genetic variation (*V*_*A*_) for that trait. Furthermore, our analyses of differentiation in neutral marker loci (F_*st*_) indicate that *V*_*A *_is less than expected on the basis of genetic drift alone, and thus, a lower *V*_*A *_in insular populations could be caused by natural selection.

**Conclusion:**

We believe that different selection pressures (e.g. higher intraspecific competition) in an insular environment might favour larger offspring size in small mammals. Island selection for larger offspring could be the preliminary mechanism in a process which could eventually lead to a smaller litter size and lower reproductive effort frequently found in insular vertebrates.

## Background

Population genetics models [1] emphasise the importance of different stochastic processes related to geographical isolation, such as the founder effect and genetic drift, on the differentiation of small populations. Severe reductions of genetic variability and population size are suggested to favour drift and constrain natural selection [2]. However, recent studies [3,4] have shown that natural selection could be the dominant diversifying agent in the evolution of quantitative traits. Irrespective of whether we consider natural selection or random drift as the major causative agent in evolution, together they may allow populations on isolated islands to evolve a collection of traits that distinguish them from their mainland relatives.

Differences between mainland and island populations of mammals have often been referred to as the Island rule or Island Syndrome [5-11]. The most familiar pattern on islands is the evolution of larger-bodied species towards a smaller size and smaller-bodied species towards a larger size [12]. In small mammals, the pattern also includes reduced reproductive output, higher survival rate, and differences in behaviour (see reviews in [9,13]). Recently the generality of the island rule has been criticized by Meiri and colleagues [14,15]. According to their phylogenetic analyses, the increase in body size might only hold true in some mammalian groups (e.g. murid rodents). They argued that earlier reviews were biased by a few extreme examples in some mammalian groups (e.g. elephants), and these reviews might have ignored many examples where body size has not changed. Furthermore, it has been suggested [15,16] that future studies should be focused more clearly on the possible differences in natural selection caused by island characters (size and isolation) [17], ecological mechanisms (e.g. predation rate and inter/intraspecific competition) [18] and species specific mechanisms (e.g. evolutionary constraints caused by additive genetic variation).

Here we focused on the possible genetic differentiation in morphological and life-history traits between insular and mainland populations of a small mammal, the bank vole *Myodes glareolus*. Our previous studies have indicated a large additive genetic variation in reproductive traits (e.g size and number of offspring) in the mainland population of our study species [19]. Furthermore, we have shown that the rapid selection caused by intraspecific competition can significantly regulate the proportion of genetic reproductive tactics (high or low reproductive effort) in mainland populations [20]. In the present study, we tested the hypothesis that the selection for reproductive tactics might differ in an insular environment, causing genetic differentiation of insular populations from mainland ones. We also tested the relative importance of natural selection and random drift on genetic differentiation. In these analyses fitness-related additive genetic variation was compared to neutral genetic variation (neutral genetic markers) [21].

## Results

**Figure 1:**
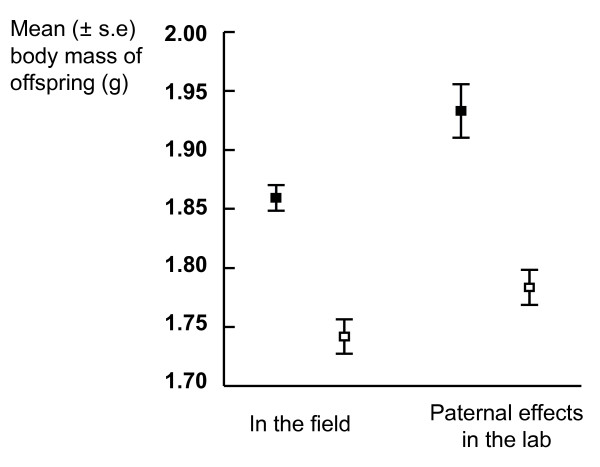
**Island offspring were significantly heavier than mainland ones in the field (see statistics in Table 1)**. In further lab analyses, males originating from island populations fathered significantly heavier offspring than mainland fathers (paternal effects) (see statistics in Table 2). (■, island; □, mainland).

We observed neither morphological differentiation in the breeding females, nor a significant difference in their reproductive effort between insular and mainland populations of the bank vole. By contrast, females that originated from the islands produced significantly larger offspring than those from the mainland (Fig 1, Table 1), indicating either environmental or genetic responses of mothers to the insular environment. The size of island or distance to mainland did not affect the breeding characters of insular females (see Additional file 1).

**Table 1:** Characteristics (mean ± SE) of breeding females from mainland and island populations.

	island (n = 51)	mainland (n = 33)	F_ndf, ddf_	p-value
Body mass of offspring (g)	1.85 ± 0.01	1.74 ± 0.02	4.54_1,23.3_	0.044
Litter size	5.0 ± 0.2	5.6 ± 0.3	3.09_1,82_	0.083
Litter mass (g)	9.25 ± 0.37	9.74 ± 0.44	0.813_1,80_	0.370
Reproductive effort (1)	0.76 ± 0.03	0.81 ± 0.04	1.20_1,82_	0.277
Reproductive effort (2)	0.40 ± 0.02	0.42 ± 0.2	1.20_1,29.1_	0.392
Post-partum head width of mother (mm)	13.2 ± 0.1	13.3 ± 0.1	0.40_1,33.9_	0.529
Post-partum body mass of mother (g)	23.2 ± 0.4	23.2 ± 0.4	0.02_1,32.1_	0.896

Only the test statistics of origin are presented from the mixed model analyses (SPSS 14.0). Origin of mother was used as a fixed factor and population as a random factor in the analyses. Mother was also used as a random factor in the analysis of offspring body mass. Reproductive effort (1) = *L *× *M*_*o*_^0.75^/*M*_*m*_^0.75^, and Reproductive effort (2) = *L *× *M*_*o*_/*M*_*m*; _where *L *is litter size; *M*_*o *_is mean pup mass at birth, and *M*_*m *_is weight of the female after delivery. *ndf *= numerator degrees of freedom, *ddf *= denominator degrees of freedom.

As the phenotypic differences in neonate size do not necessarily imply micro-evolutionary differentiation, the genetic basis of offspring size was further analysed in the laboratory using paternal half sib analyses. Males originating from island populations fathered significantly heavier offspring at birth than mainland fathers (Fig 1, Table 2), when both were mated to a common stock of females. Moreover, the analyses indicated significant additive genetic variance only among the mainland fathers (*V*_*A *_± S.E. = 0.047 ± 0.020 and *h*^2 ^± S.E = 0.96 ± 0.41), whereas genetic variance was zero or very low among island fathers (Table 3). Heritability estimates differed significantly between the mainland and island fathers (t = 3.67, df = 26, *P *< 0.002) (Table 3).

**Table 2:** Mixed Model Analyses (SPSS) for differences in body mass of offspring fathered either by mainland or island males.

Source	ndf	ddf	F	Wald Z	P
Origin of sire	1	6.8	10.488		0.015
Sire	27	48.3	1.828		0.033
Dam (sire)	48	278	14.631		< 0.001
Population				0.240	0.810

Origin of sire, sire and dam (within sire) were used as fixed factors and population as a random factor. *ndf *= numerator degrees of freedom, *ddf *= denominator degrees of freedom.

**Table 3:** Genetic basis of the birth mass of offspring sired by fathers from two different origins.

	Source	df	MS	F	P	V_P_	V_A _± S.E.	h^2 ^± S.E.
Mainland								
	Sire	7	0.298	2.384	0.047	0.049	0.047 ± 0.020*	0.96 ± 0.41*
	Dam (sire)	28	0.140	13.010	< 0.001			
	Error	171	0.011					
Island								
	Sire	5	0.328	1.676	0.186	0.081	0.012 ± 0.012^n.s.^.	0.14 ± 0.24^n.s.^.
	Dam (sire)	19	0.294	16.918	< 0.001			
	Error	107	0.017					

Phenotypic variances (V_P_), additive genetic variances (V_A_) and heritabilities (h^2^) of body mass were estimated by half-sib analyses from the variance components among sires separately for the different environments. Standard errors of V_A _and h^2 ^were estimated using the formula in [59]. The effect of population (P > 0.39 in both origins) was included to the models as a random factor.* *P *< 0.05, n.s.= non-significant.

The relative importance of natural selection and random drift on offspring size divergence can be tested by comparing among-island differences based on additive genetic variation and measures based on neutral marker genes (*F*_*st*_) [21]. Here the island populations were different according to neutral markers (*F*_*st *_± *S.E. *= 0.177 ± 0.021; *P *< 0.001) but not according to the additive genetic variance (*V*_*A *_≈ 0) (Table 3). This means that the *V*_*A *_was less than expected on the basis of genetic drift alone. Mainland populations (localities) did not differ according to neutral markers (*F*_*st *_± *S.E. *= 0.008 ± 0.006; n.s.). F_*st *_values differed significantly between mainland and island populations (*P *= 0.001).

## Discussions and conclusion

The present results are in agreement with earlier suggestions that offspring size might be the first life-history characteristic to evolve in insular populations of vertebrates [22,23]. Large neonate size can be a local adaptation to an insular environment, where many ecological selection pressures, e.g. intra- and inter-specific competition and predation, differ from larger mainland populations [9]. Here we could not directly test the biological significance of larger offspring size (0.11 g difference between the island and mainland populations), but according to our previous research, the increase in female offspring size from 1.74 g to 1.85 g may have an important impact on the future fitness of bank vole offspring [19]. For example, the age of first breeding would decreased by 5 days, calculated by a linear regression model (y = -46.11x + 181; Fig 1b in [19]), and the probability of breeding was increased from 0.68 to 0.78, calculated by a logistic regression model (ln(y/1-y) = 4.87x - 7.70; Fig. 2a in [19]).

The breeding density of territorial bank vole females seems to be lower in open populations (approx. 10 females/ha maximum) [24] compared to the artificially enclosed populations (e.g. over 20 females/ha) [25]. If intra-specific competition between breeding females is similarly increased on (enclosed) islands, it could be an important selective force for the larger offspring size at birth. In general, the precise mechanisms of selection and when intra-specific competition may favour the fitness of larger offspring or adult size have not yet been tested in insular populations [18]. There exists only indirect evidence that competition is lower on larger islands, decreasing selection for body size in mammals [17]. Here we did not find any effect of island area or isolation on the measured morphological or life-history traits. We suggest that compared to the earlier studies, the present islands were relatively small and variation in size might be too low to find the significant effects of island characters. However, our findings are in agreement with most of the earlier studies, which do not support the importance of island characters for the differentiation of insular individuals from their mainland descendants [26-30].

The phenomenon to produce large offspring can also be linked to the genetic dispersal tactics of individuals [22,31]. The individuals which are more prone to take risks, e.g. by dispersing over large open ice to islands, might also genetically differ according to many other traits besides offspring size. The change in offspring size could then be a by-product of selection on other traits. This possibility cannot be ruled out before knowing more about population genetics and the behaviour of individuals (e.g. extinctions, dispersal and mutation rates) in our island system. Even the loss of additive genetic variation in offspring body mass that we have shown here can be explained by non-adaptive genetic processes, e.g. random drift. However, our analyses of differentiation in neutral marker loci (F_st_) indicate that *V*_*A *_is less than expected on the basis of genetic drift alone [21]. Here, we were not able to analyse additive genetic variance or variance in neutral markers within single islands, as our estimates of additive genetic variance were based on a few individuals per location. Therefore, comprehensive comparisons of differentiation in neutral marker loci and additive genetic variation in quantitative traits are still lacking [21].

In contrast to the island populations, additive genetic variation in offspring body mass has been observed in mainland populations [[19] and here]. Additive genetic variation was also higher than expected by the variation in neutral markers. Similarly, a large additive genetic variation has also been show in several other life-history traits in different systems [32-34]. In agreement with our findings, the additive genetic variation is also usually higher than the variation in neutral markers [21]. Additive genetic variation in life-history traits can be maintained by a trade-off (negative genetic correlation) between two traits [35], in this case between the size and number of offspring [19], especially when natural selection favours one trait under current conditions and another at a later date [36]. Strong annual and multi-annual density fluctuations (cyclicity) are suggested to maintain additive genetic variation and even genetic polymorphism in life-history traits, particularly in rodent populations [37]. In fact, our recent findings with the bank vole indicate that density- and negative frequency-dependent selection favour the genetically different allocation tactics between the size and number of offspring [20].

We supposed that if density fluctuations and other ecological parameters related to them are more stable in insular environments, selection for large offspring size could also be stable long term. Theoretically, strong selection might decrease additive genetic variation found in our islands [34,38,39]. Moreover, selection for large offspring size might simultaneously decrease litter size [19]. A future goal would be to show whether large neonate size is an adaptation to insular environments, and also how genetic differentiation in this particular trait is related to other life-history and behavioral traits (e.g. litter size, reproductive effort, adult size, longevity, disperal) as well as their evolution.

## Methods

### Study species

The bank vole is a common mammal in coniferous forests of northern Europe [40]. The breeding period in central Finland lasts from May to September [25]. Pregnancy lasts for 19–20 days and pups are weaned until the age of three weeks [41]. In addition to remarkably large phenotypic [25] and additive genetic variation [19] in litter size (2–10) and offspring size (1.3–2.5 g), a trade-off (i.e. both negative phenotypic and genetic correlations) also exists between these traits [19]. Furthermore, a larger size at birth [19,24] and at weaning [41] increases the probability of maturation (i.e. breeding in summer they are born) in juvenile females. Reproducing bank vole females are territorial, while home ranges of males and non-breeding individuals overlap [42-44]. The density of breeding females is limited due to their territoriality [45].

### Field sample

The study was carried out in central Finland (62° 37'N, 26° 20'E). The data are based on 898 individuals caught from 37 islands (0.12–70 ha) in lake Konnevesi and from 20 mainland localities within 5 km of the lake during the summer 1999. Pregnant females were caught from 20 islands and all mainland areas, and thus only these populations were included in the present study. The shortest distance between neighbouring study islands varied between 50 to 500 m and the mean distance from islands to the mainland was 631 m (S.E. = 75 m). Dispersal between islands during summer is very low, indicated by a separate study during the autumn where we recaptured 106 island individuals (three to five months from the first capture); none of these individuals left their home islands. The mainland trapping areas were sufficiently far apart (mean ± S.E = 832 ± 129 m, range 300 – 2 000 m) to decrease dispersal between different mainland localities. We were not able to estimate dispersal rate between mainland localities, but according to earlier studies [25], it should be very low especially among territorial breeding females. The individuals were caught using Ugglan multiple-capture live-traps. On the smaller islands (< 3.5 ha), trap lines were set at c. 20 m intervals (25 traps/ha). On the larger islands (> 3.5 ha) and in the mainland localities, individuals were trapped using the small quadrat sampling method (modified from [46]: each quadrat area (side = 15 m) contained four trap sites (4 traps/area). Pre-baited traps were left open for two nights, after which they were set and checked over three consecutive days. Trappings were carried out from early May to the end of the breeding season in September.

All trapped voles were taken into the laboratory where each individual was sexed, weighed to the nearest 0.1 g, and measured for maximum head width to the nearest 0.1 mm with a digital calliper [47]. Males and non-pregnant females were then released back to the field. 51 pregnant females from 20 islands and 33 pregnant females from 20 mainland localities were kept in the laboratory prior to producing a litter [41]. Immediately after birth, pups were weighed with an electronic balance to the nearest 0.01 g, and width of head was measured using a stereomicroscope. The mothers were released with their pups at their point of capture [41]. The proportion of breeding females did not differ between the islands (21.2%, *n *= 241) and mainland (26.8%, *n *= 123)(*G *= 1.45, *df *= 1, *P *= 0.229).

The reproductive effort of females was estimated using two formulas. First, we used the formula:*RE(1) *= *L *× *M*_*o*_^0.75^/*M*_*m*_^0.75^, where *L *is litter size; *M*_*o *_is pup mass at birth, and *M*_*m *_is weight of the female after delivery [22,48]. In this formula, energy requirements to produce offspring is calculated relative to the allometric requirement of the mother (assuming standard metabolism increases to the 0.75 power of mass for mammals) [48-51]. The mothers were released with their pups at their point of capture [41]. Since the theoretical and empirical basis of 0.75 scaling is still under debate in the literature of animal metabolism (see e.g. [52,53]), we also used a more simple formula: *RE(2) *= *L *× *M*_*o*_/*M*_*m*_, where litter mass was divided by mother body mass.

### Analyses of quantitative genetics and neutral markers

The genetic basis of offspring characteristics was analyzed in the laboratory. In the analyses, we compared the effect of male origin (island/mainland) on the characteristic of their offspring. Bank vole males do not rear their offspring and male quality does not affect the amount of maternal care [54], hence we can assume that the genetic analyses are not biased by covariances between non-genetic maternal effects and genetic paternal effects.

We mated a random sample of males from the mainland and island populations with two to three randomly chosen females that originated from a separate lab colony (Fig. 1, Table 2). The females mated with the island males did not differ from the females mated with mainland males (head width: (*t *= 0.255, *df *= 73, *P *= 0.800; body mass: *t *= 1.651, *df *= 73, *P *= 0.103). Body mass and head width of the males that originated from islands did not differ from the males that originated from the mainland (Mixed model analyses (SPSS);origin fixed and population random factor; head width: F_1,15.7 _= 1.21, *P *= 0.287; body mass: F_1,10.9 _= 1.13, *P *= 0.311). To obtain an estimate of heritability (h^2^) and additive genetic variance (*V*_*A*_) for the body mass of offspring in the mainland and island populations, we performed standard half-sib analyses (Table 3). The analyses included 16 sires, 44 dams and 216 progeny from mainland populations and 12 sires, 31 dams and 140 progeny from island populations. We were only able to include one to three males per island or mainland location to the analyses, so our estimates of genetic variance indicate the variance among the whole island system, not variance within single islands.

Individuals were genotyped with six microsatellite loci, which are highly variable in the bank vole [55]. To obtain comparable analyses, we used mature males from the same islands (62 males from 9 islands) and mainland localities (75 males from 10 areas) as we used in the analyses of quantitative genetics. An estimate of population structure was obtained using *F*_*st *_[56], calculated using FSTAT ver 2.9.3 [57]. Standard error was obtained with Jacknifing over loci. The significance of population differentiation was tested by log-likelihood *G*-statistics and the test was based on 1000 randomization of genotypes within samples [58].

## Authors' contributions

TM, HH, EH, EK, RS and PS planned the study and performed the field trappings. TM performed the analyses of quantitative genetics. AG was responsible for the analyses of molecular genetics.

## Supplementary Material

Additional file 1 **Mixed Model Analyses (SPSS) for the effects of island size and island distance to mainland on the breeding characters of insular females.** Size of island and distance to mainland were used as fixed factors (covariates) and population as a random factor in the analyses. *ndf *= numerator degrees of freedom, *ddf *= denominator degrees of freedom. Click here for file
