# Supplementary material for: Island selection on mammalian life-histories: genetic differentiation in offspring size
Source: BMC Evol Biol. 2008 Oct 27;8:296. doi: 10.1186/1471-2148-8-296 (PMC2584046; doi:10.1186/1471-2148-8-296)
Supplement: Additional file 1 — Mixed Model Analyses (SPSS) for the effects of island size and island distance to mainland on the breeding characters of insular females. Size of island and distance to mainland were used as fixed factors (covariates) and population as a random factor in the analyses. ndf = numerator degrees of freedom, ddf = denominator degrees of freedom. [file 1471-2148-8-296-S1.doc]

Mixed Model Analyses (SPSS) for the effects of island size and island distance to mainland on the breeding characters of insular females. Size of island and distance to mainland were used as fixed factors (covariates) and population as a random factor in the analyses. *ndf =* numerator degrees of freedom, *ddf* = denominator degrees of freedom.

|  | Source | *ndf* | *ddf* | F | *P* |
| --- | --- | --- | --- | --- | --- |
| Mean body mass of offspring | Size of island | 1 | 16.4 | 0.83 | 0.375 |
|  | Distance to mainland | 1 | 14.3 | 0.54 | 0.474 |
|  |  |  |  |  |  |
| Litter size | Size of island | 1 | 48.0 | 0.26 | 0.614 |
|  | Distance to mainland | 1 | 48.0 | 0.01 | 0.935 |
|  |  |  |  |  |  |
| Litter mass | Size of island | 1 | 46.0 | 0.00 | 0.956 |
|  | Distance to mainland | 1 | 46.0 | 0.15 | 0.701 |
|  |  |  |  |  |  |
| Reproductive effort | Size of island | 1 | 16.0 | 0.45 | 0.514 |
|  | Distance to mainland | 1 | 13.1 | 0.01 | 0.920 |
|  |  |  |  |  |  |
| Post-partum head width of mother | Size of island | 1 | 10.7 | 0.26 | 0.619 |
|  | Distance to mainland | 1 | 10.8 | 2.47 | 0.145 |
|  |  |  |  |  |  |
| Post-partum body mass of mother | Size of island | 1 | 12.3 | 0.61 | 0.448 |
|  | Distance to mainland | 1 | 13.3 | 0.22 | 0.650 |
